# A novel radiographic analysis system for subaxial cervical spine pedicle screw placement

**DOI:** 10.1186/s13018-023-03999-9

**Published:** 2023-08-08

**Authors:** Baozhi Ding, Tangjun Zhou, Hui Ma, Jie Zhao

**Affiliations:** 1 Shanghai Key Laboratory of Orthopaedic Implants, Shanghai, People’s Republic of China; 2 Department of Orthopaedic Surgery, Shanghai Ninth People’s Hospital, Shanghai Jiao Tong University School of Medicine, Shanghai, 200011 People’s Republic of China

**Keywords:** Accurate, Cervical spine, CT scan, Pedicle screw, Placement

## Abstract

**Background:**

Precise pedicle screw placement of the subaxial cervical spine is difficult. Not every hospital is equipped with a guidance system that can provide effective help. Computed tomography (CT) scanning is almost a routine preoperative examination for cervical spine surgery in all hospitals. Appropriate measurement and analysis of the CT images could assist optimal cervical pedicle screw placement. The purpose of this study is to propose a new and universal method using computed tomography (CT) morphological parameters analysis to assist optimal cervical pedicle screw placement from C3 to C7.

**Methods:**

A localization system with six parameters was designed based on preoperative CT reconstruction to guide subaxial cervical spine pedicle screw placement. The six parameters were distance from the starting point to the midline [D1], distance from the starting point to the lower edge of the inferior articular process [D2], transverse section angle [TSA], sagittal section angle [SSA], pedicle width [PW], and pedicle height [PH]. The six parameters were analyzed in 53 participants.

**Results:**

Combining D1 and D2 could localize the entrance of the pedicle screw, and we concluded that D1 and TSA and D2 and SSA could be a new standard for determination of the transverse and sagittal orientation of the pedicle screw. The six parameters were closely related to the patient’s gender, height, and weight. PH and PW were linearly correlated and could guide selection of the appropriate pedicle screw. SSA was an independent parameter of the relevant vertebral body, and changes in SSA had nothing to do with the curvature or posture of the cervical spine.

**Conclusions:**

Understanding and applying the six-parameter localization system are essential for achieving accurate and optimal pedicle screw placement in subaxial cervical spine, regardless of cervical sagittal alignment.

## Introduction

The subaxial cervical pedicle screw placement technique has been widely used in clinical practice since it was first described by Abumi [1] in 1994. However, this delicate surgical procedure is associated with a risk of major neurovascular injury. The cervical pedicles are slim and small, with great variation in their directions; therefore, accurate pedicle screw placement is essential to prevent spinal cord, nerve root, and vertebral artery iatrogenic injury [2, 3]. Precise pedicle screw placement requires information regarding at least two important anatomical concepts: the pedicle screw insertion point and the pedicle screw route, including direction and depth. The establishment of an insertion point is the first and key step to perfect pedicle screw placement. Further, the insertion route in accordance with the axis of the pedicle screw can make maximum use of the pedicle coronal and sagittal diameter and is the safest route [4].

Freehand pedicle screw placement mainly depends on the surgeon’s judgment of relevant anatomical landmarks during the operation [5, 6]. This localization method is dangerous and difficult however, especially for inexperienced surgeons, because the anatomical features vary with race, gender, height, weight, deformity, and degeneration. Therefore, the surgeon’s experience and feel are critical in these situations.

Alternatively, a guidance system such as O-arm-based three-dimensional (3D) navigation, 3D model, navigation templates, Doppler donography, robotic guidance system [7–12], or augmented reality-based navigation [13, 14] is needed to prevent placement failure. The use of these resources is limited because of their high cost and steep associated learning curve however, and they cannot be widely applied in all kinds of hospitals [15, 16]. Fortunately, computed tomography (CT) scanning has become a routine preoperative examination in spinal surgery patients. Moreover, CT reconstruction is accurate, and a 3D (coronal sagittal, and cross-sectional) surface can be easily obtained at any angle to meet the requirements of personalized measurement [17, 18].

The current study investigated the establishment of a new subaxial cervical pedicle radiographic system utilizing preoperative CT scanning and reconstruction, which could determine the pedicle’s detailed morphology and has the advantages of individualized application, high accuracy, and easy identification.

## Patients and methods

### Study participants

Fifty-three patients (31 men, 22 women) with different cervical spine diseases were enrolled in the study. Baseline characteristics of the participants are shown in Table 1. The inclusion criteria were performance of supine CT (Philips ICT) of the cervical spine, and image data analysis in the Image Clinical Application and Platform and 3-matic software (Materialise, Belgium). Patients with pedicle deformity and destruction of the cervical spine because of spinal tumor, infection, or trauma were excluded. The study was approved by the institutional ethics review board. Written informed consent was obtained from each patient.

**Table 1:** Baseline of the participants

Age (yrs ± SD)		59.2 ± 9.0	Sex (male/female)		31/22
Parameters	C3	C4	C5	C6	C7
Six parameters from L1-L5 (Mean ± SD)					
PW(mm)	6.25 ± 0.87	6.22 ± 0.91	6.37 ± 081	6.74 ± 0.91	7.47 ± 1.12
D1(mm)	21.74 ± 1.65	22.66 ± 1.95	23.51 ± 1.97	22.84 ± 2.11	20.96 ± 2.40
TSA(°)	46.29 ± 5.37	48.78 ± 5.31	47.40 ± 5.35	42.35 ± 5.48	34.31 ± 7.04
PH(mm)	7.29 ± 1.03	7.89 ± 1.08	7.21 ± 0.94	7.33 ± 1.09	8.19 ± 1.20
D2(mm)	9.78 ± 2.04	9.52 ± 2.08	10.02 ± 2.04	10.17 ± 2.19	10.23 ± 2.31
SSA(°)	102.10 ± 9.13	100.40 ± 10.31	90.82 ± 8.12	88.21 ± 8.83	95.65 ± 9.50
C3-7 Cobb° = 5.0 ± 10.6°					

PW = pedicle width; D1 = the distance from the starting point to midline; TSA = transverse section angle; PH = pedicle height; D2 = the distance from the starting point to the lowest point; SSA = sagittal section angle

### Imaging measurement

The axis of the pedicle was defined as the intersection line of the equally divided transverse plane (plane B) and vertical plane (plane A) of the pedicle. The intersection point of the axis on the cortex of the posterior end of the pedicle is the starting point (SP). LP is the lowest point on the lower edge of the inferior articular process on the line where plane A intersects the posterior bone surface. SP was used as the best entry point of the pedicle screw, and the axis of the pedicle was used as the best insertion route.

**Fig. 1:**
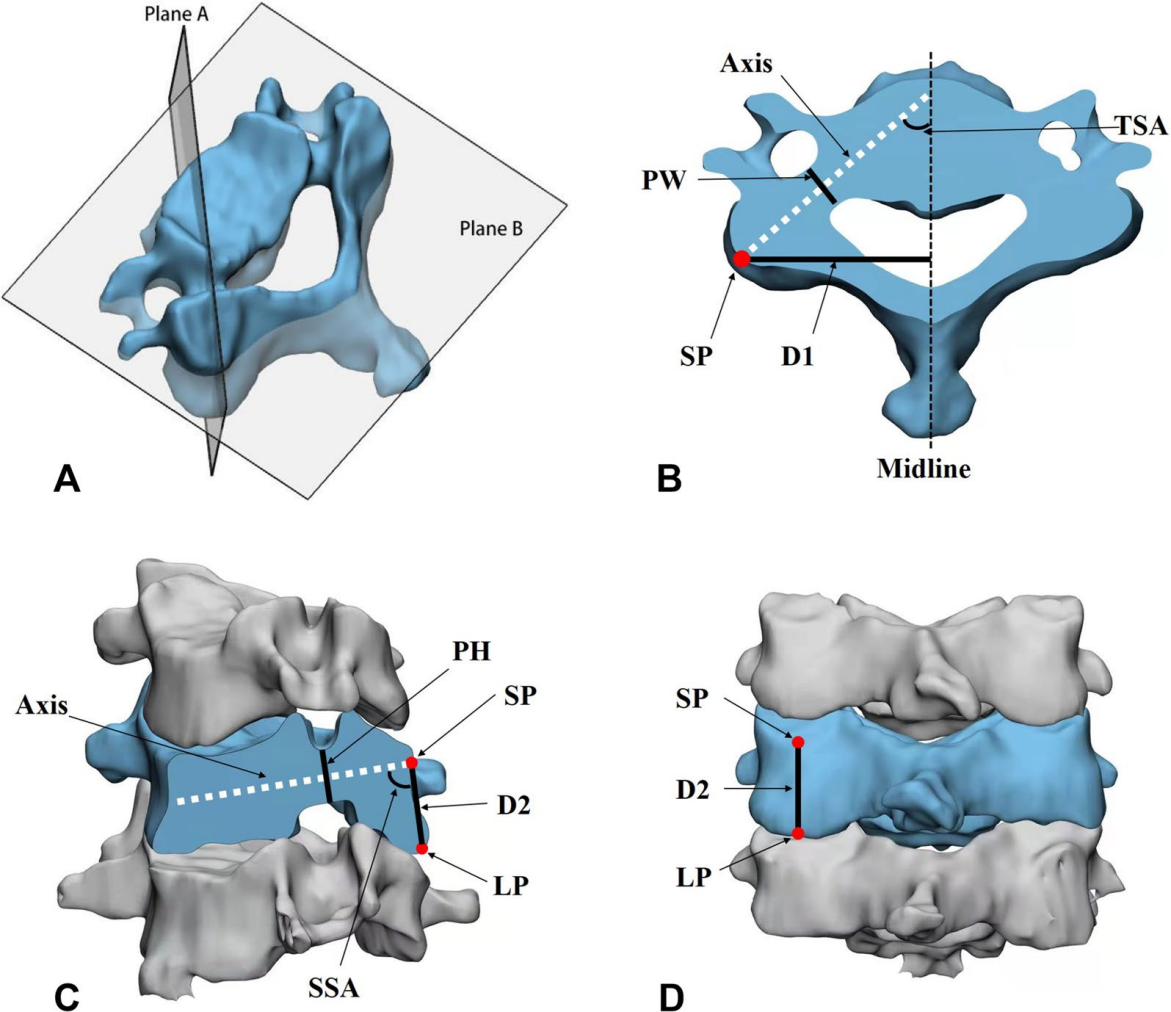
Schematic diagram of parameter measurements. **A** Three-dimensional reconstruction of a cervical vertebral body from a CT scan, with the pedicle equally divided by plane A and plane B; **B** Transverse section of the vertebral body after being cut by plane B. The white dotted line is the axis of the pedicle; SP = starting point; PW = pedicle width; D1 = distance from SP to midline; TSA = transverse section angle; **C** Sagittal section of the vertebral body after being cut by plane A. LP = lowest point; PH = pedicle height; D2 = straight-line distance from SP to LP; SSA = sagittal section angle; **D** Posterior view of the vertebral body

Six parameters were measured based on CT reconstruction derived from each patient (Fig. 1). Pedicle width (PW) was the narrowest width of the pedicle in the equally divided transverse plane, which is perpendicular to the axis of the pedicle. Pedicle height (PH) was the shortest height of the pedicle in the equally divided vertical plane, which is perpendicular to the axis of the pedicle. D1 was the distance from SP to the midline of the spinous process. D2 was the straight-line distance from SP to LP. The transverse section angle (TSA) was the angle of the axis and middle line on the transverse plane. The sagittal section angle (SSA) was the angle of the axis and D2 line on the sagittal plane. Both pedicles were measured. The height and weight of each patient were recorded, changes in all six parameters from C3 to C7 were analyzed, and correlations between each of them were assessed. The system was based on CT scanning, which allowed us to obtain accurate measurement data for the pedicle with abnormal anatomical structure such as deformity or degeneration. Software measurement can reduce errors such that the accuracy of linear data reaches 0.01 mm and the angle is equivalent to 0.01°.

### Statistical analysis

All parameters were measured twice by the same observer on two different occasions and once by another observer to determine intraobserver and interobserver reliability, which was evaluated via intraclass correlation coefficients (ICCs). The reliability of intraobserver and interobserver measurements was consistent if the ICC was between 0.82 and 0.98. Measurements obtained by one observer were used in the analysis.

Measurement data were expressed as mean ± SD. The chi-square test and matched or unmatched *t*-test were used to evaluate differences between two groups. Pearson’s correlational coefficient (*r*) was used to assess correlations between variables. Statistical significance was set at *p* < 0.05. Correlation coefficients were considered clinically statistically significant if r ≥ 0.3. All data were analyzed via SPSS version 22.0 (SPSS, Chicago, IL).

## Results

### Changes in the six parameters from C3 to C7

**Fig. 2:**
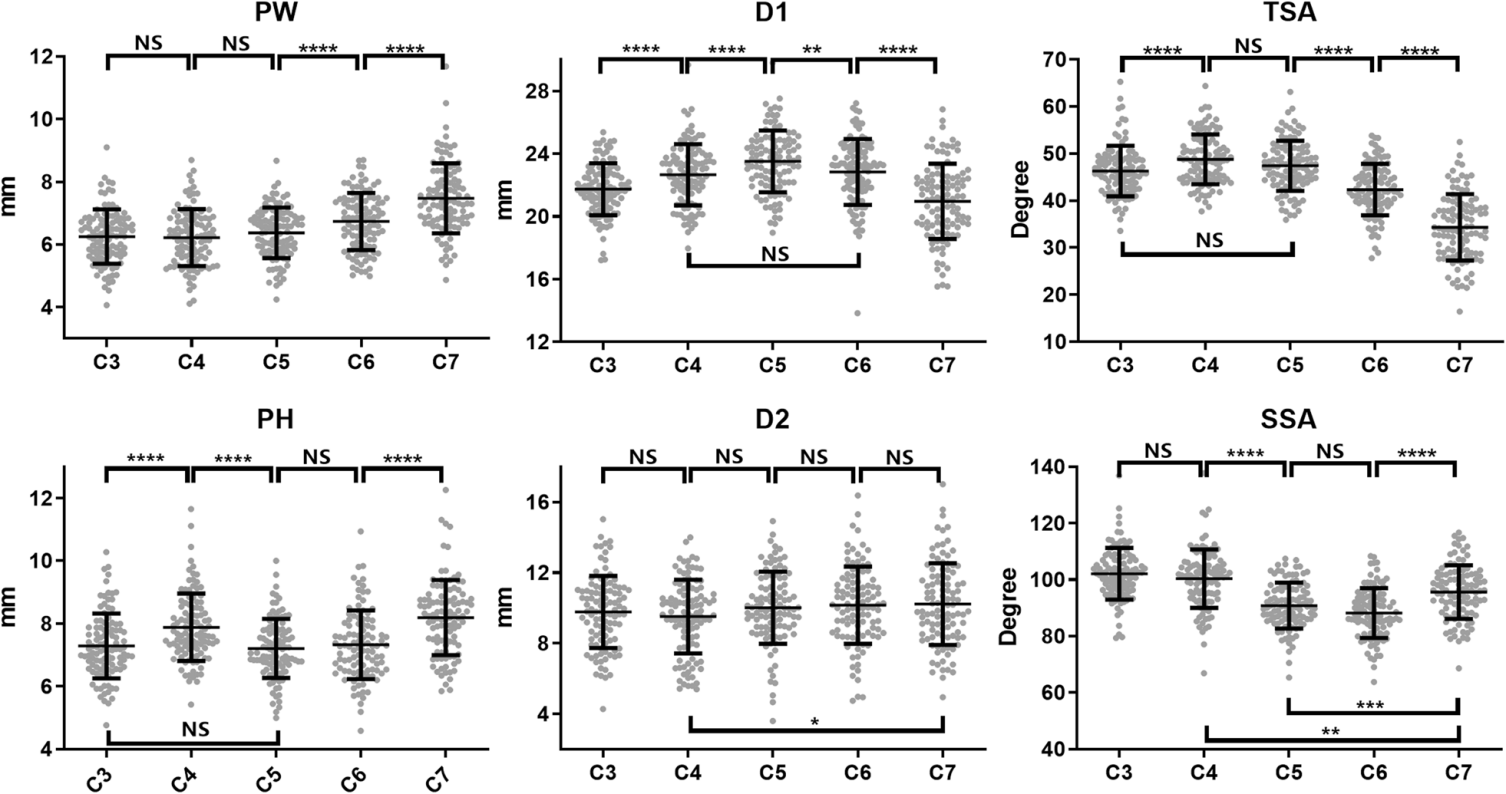
Scatter plot showing changes in the six parameters from C3 to C7. Data are presented as means ± SD. *p < 0.05, **p < 0.01, ***p < 0.001, ****p < 0.0001

There were no significant differences in any of the six parameters between both sides of the pedicles from the same cervical segment. PW gradually increased from C3 to C7. D1 gradually increased first, reached a maximum at C5, and then gradually decreased. TSA was largest at C4 then decreased gradually from C4 to C7, and TSA at C3 was similar to that at C5. The PHs of C3, C5, and C6 were low, whereas those of C4 and C7 were relatively high. D2 remained similar from C3 to C7. SSA gradually decreased from C3 to C6 and slightly increased at C7 (Fig. 2).

**Fig. 3:**
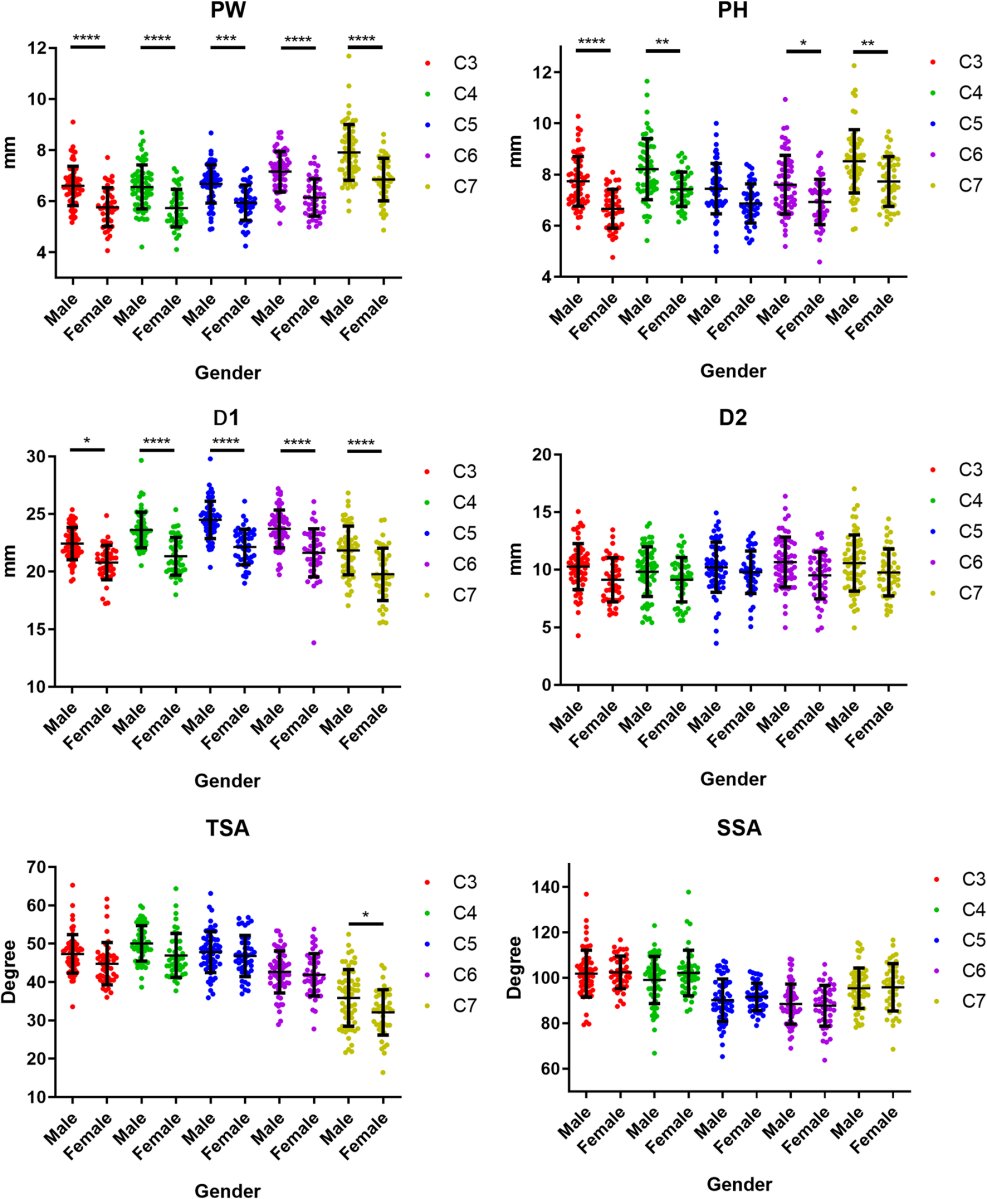
Scatter plot showing comparisons of the six parameters between men and women. Data are presented as the means ± SD. *p < 0.05, **p < 0.01, ***p < 0.001, ****p < 0.0001

With the exception that in C5 PH did not differ significantly in men and women, PW, PH, and D1 were generally greater in males than in females in all segments. There were no significant differences in D2, TSA, or SSA between males and females in any segments, except that the TSA of C7 was greater in males (Fig. 3).

### Linear correlations between PW and PH

**Fig. 4:**
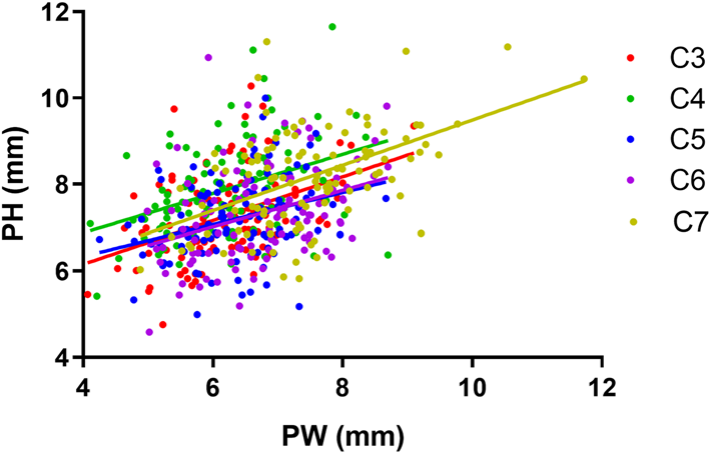
Linear regression between PW and PH

There were positive correlations between PW and PH in all cervical segments (Fig. 4). From C3 to C7 the slope and *Y*-intercept were similar (Table 2), indicating that the increase in PW has a similar effect on the increase in PH, the ellipse cross-sectional area of the pedicle with H as the long axis gradually increased from C3 to C7.

**Table 2:** Linear correlation between PW and PH, D1 and TSA

Linear correlation	Segment	Equation	*R*	Slope	*Y*-intercept	*P*
X = PW Y = PH	C3	*Y* = 0.5044*X + 4.135	0.4221	0.1062	0.6705	< 0.0001
	C4	*Y* = 0.4544*X + 5.058	0.3816	0.1079	0.6784	< 0.0001
	C5	*Y* = 0.3678*X + 4.864	0.3155	0.1085	0.6969	0.0010
	C6	*Y* = 0.4220*X + 4.482	0.3517	0.1101	0.7488	0.0002
	C7	*Y* = 0.5242*X + 4.276	0.4898	0.0915	0.6911	< 0.0001
X = D1 Y = TSA	C3	*Y* = 1.739*X + 8.497	0.5349	0.2693	5.870	< 0.0001
	C4	*Y* = 1.371*X + 17.71	0.5026	0.2313	5.260	< 0.0001
	C5	*Y* = 1.052*X + 22.68	0.3868	0.2458	5.798	< 0.0001
	C6	*Y* = 1.191*X + 15.15	0.4575	0.2269	5.205	< 0.0001
	C7	*Y* = 2.005*X − 7.720	0.6845	0.2094	4.418	< 0.0001

* means multiply by

PW = pedicle width; PH = pedicle height; D1 = the distance from the starting point to midline; TSA = transverse section angle

### Linear correlations between D1 and TSA

**Fig. 5:**
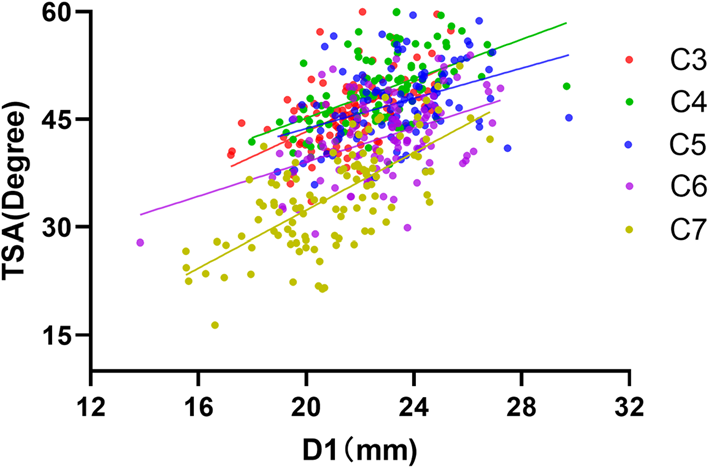
Linear regression between D1 and TSA

There were positive correlations between D1 and TSA in all cervical segments (Fig. 5), and the linear Pearson’s correlational coefficient between D1 and TSA was the highest in C7 (Table 2). Therefore, for a segment, the more externally deviated the SP the larger the TSA, and conversely the more internally deviated the SP the smaller the TSA.

### Linear correlations between weight and PW, weight and PH, weight and D1, height and PW, height and PH, and height and D1

**Fig. 6:**
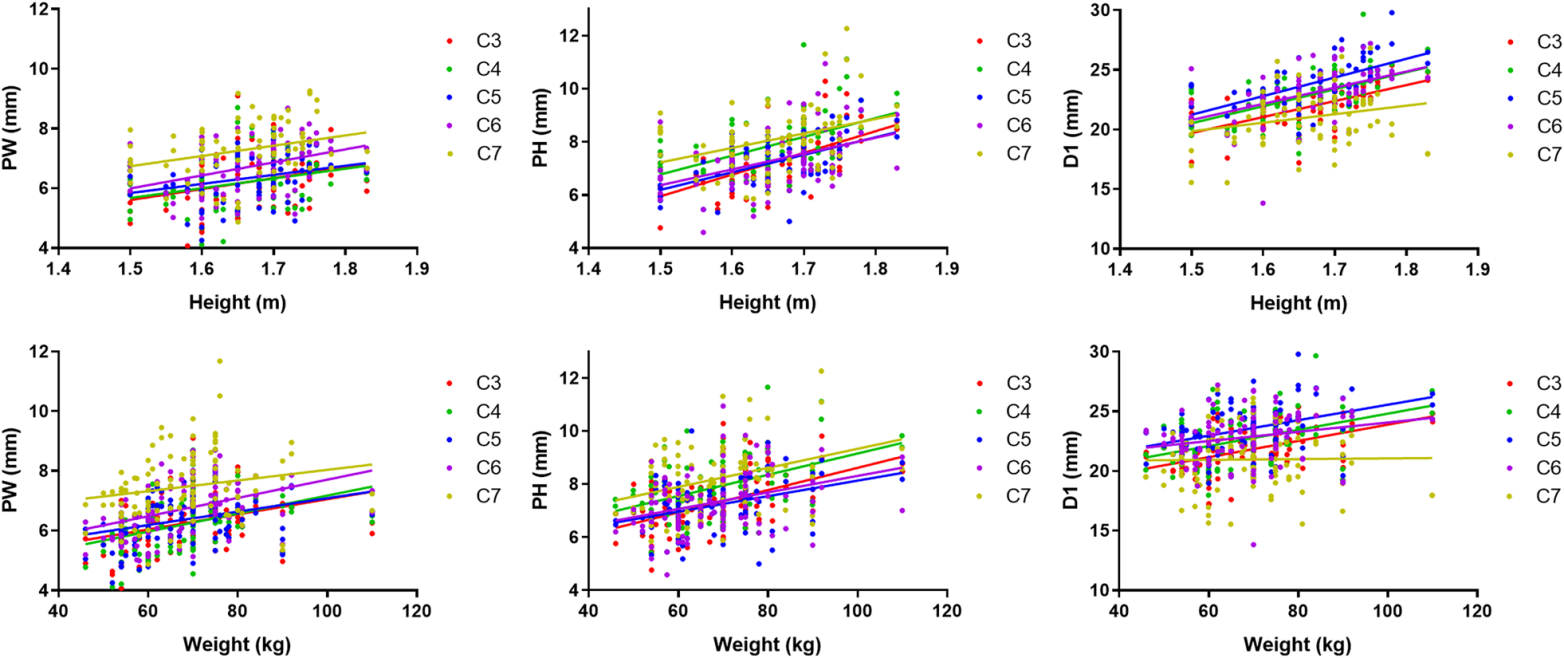
Linear correlations between weight and PW, weight and PH, weight and D1, height and PW, height and PH, and height and D1

Both height and weight were positively correlated with PW, PH, and D1 (Fig. 6), indicating that patients with greater height and weight had larger pedicle cross sections, and the SP was more externally deviated. The linear Pearson’s correlational coefficient between height, weight and PH, D1 was better than that of PW, and the closer to the upper segment from C7–C3, the better the linear fit was (Tables 3 and 4). From C3 to C7 all slopes exhibited an upward trend, and the slope value was the largest at C7, indicating that increases in height and weight had the greatest impact on increases in PW, PH, and D1 in the C7 segment.

**Table 3:** Linear correlation between Height and PW, Height and PH, Height and D1

Linear correlation	Segment	Equation	*R*	Slope	*Y*-intercept	*P*
X = Height Y = PW	C3	*Y* = 3.674*X + 0.09572	0.3198	1.265	2.103	0.0049
	C4	*Y* = 3.274*X + 0.7565	0.2857	1.277	2.123	0.0124
	C5	*Y* = 3.041*X + 1.275	0.3060	1.100	1.828	0.0072
	C6	*Y* = 4.367*X − 0.5627	0.3953	1.179	1.960	0.0004
	C7	*Y* = 3.437*X + 1.579	0.2928	1.305	2.169	0.0103
X = Height Y = PH	C3	*Y* = 8.169*X − 6.304	0.6107	1.232	2.047	< 0.0001
	C4	*Y* = 7.081*X − 3.858	0.4969	1.438	2.390	< 0.0001
	C5	*Y* = 6.603*X − 3.720	0.5611	1.132	1.882	< 0.0001
	C6	*Y* = 5.959*X − 2.580	0.4253	1.474	2.450	0.0001
	C7	*Y* = 5.460*X − 0.9711	0.3589	1.650	2.744	0.0015
X = Height Y = D1	C3	*Y* = 13.40*X − 0.3939	0.6144	2.000	3.325	< 0.0001
	C4	*Y* = 14.26*X − 0.8643	0.5783	2.338	3.886	< 0.0001
	C5	*Y* = 15.65*X − 2.238	0.6209	2.296	3.817	< 0.0001
	C6	*Y* = 13.55*X + 0.4598	0.4749	2.920	4.854	< 0.0001
	C7	*Y* = 7.138*X + 9.145	0.2425	3.320	5.519	0.0348

* means multiply by

PW = pedicle width; PH = pedicle height; D1 = the distance from the starting point to midline

**Table 4:** Linear correlation between Weight and PW, Weight and PH, Weight and D1

Linear correlation	Segment	Equation	*R*	Slope	*Y*-intercept	*P*
X = Weight Y = PW	C3	*Y* = 0.02532*X + 4.522	0.3501	0.00664	0.4612	0.0002
	C4	*Y* = 0.03014*X + 4.157	0.3984	0.00680	0.4726	< 0.0001
	C5	*Y* = 0.02250*X + 4.834	0.3341	0.00622	0.4322	0.0005
	C6	*Y* = 0.03062*X + 4.643	0.4022	0.00683	0.4746	< 0.0001
	C7	*Y* = 0.01784*X + 6.250	0.1911	0.00898	0.6240	0.0497
X = Weight Y = PH	C3	*Y* = 0.04194*X + 4.420	0.4855	0.00740	0.5143	< 0.0001
	C4	*Y* = 0.04006*X + 5.143	0.4447	0.00791	0.5496	< 0.0001
	C5	*Y* = 0.02922*X + 5.208	0.3722	0.00714	0.4962	< 0.0001
	C6	*Y* = 0.03112*X + 5.196	0.3409	0.00841	0.5847	0.0004
	C7	*Y* = 0.03596*X + 5.732	0.3600	0.00914	0.6348	0.0002
X = Weight Y = D1	C3	Y = 0.06820*X + 17.07	0.4944	0.01176	0.8166	< 0.0001
	C4	Y = 0.06795*X + 18.01	0.4182	0.01447	1.005	< 0.0001
	C5	Y = 0.06480*X + 19.07	0.3947	0.01479	1.028	< 0.0001
	C6	Y = 0.03891*X + 20.18	0.2212	0.01682	1.168	0.0227
	C7	Y = 0.002969*X + 20.76	0.0148	0.01967	1.366	0.8803

* means multiply by

PW = pedicle width; PH = pedicle height; D1 = the distance from the starting point to midline

### Linear correlations between Cobb angle and PH, Cobb angle and D2, and Cobb angle and SSA

**Fig. 7:**
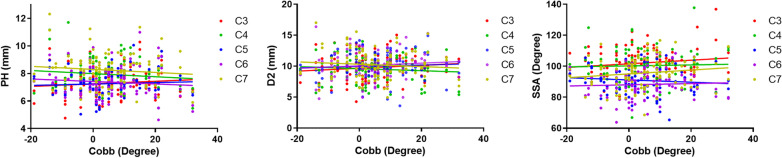
Linear correlations between Cobb angle and PH, Cobb angle and D2, and Cobb angle and SSA

Cobb angle was not significantly correlated with PH, D2, or SSA (Fig. 7, Table 5), indicating that the application of these sagittal parameters does not need to consider curvature or posture of the cervical spine.

**Table 5:** Linear correlation between Cobb and PH, Cobb and D2, Cobb and SSA

Linear correlation	Segment	Equation	*R*	Slope	*Y*-intercept	*P*
X = Cobb Y = PH	C3	*Y* = 0.009390*X + 7.245	0.0959	0.00956	0.1104	0.3283
	C4	*Y* = − 0.01124*X + 7.938	0.1100	0.00995	0.1150	0.2616
	C5	*Y* = 0.005081*X + 7.183	0.0571	0.00871	0.1006	0.5612
	C6	*Y* = − 0.008983*X + 7.368	0.0867	0.01012	0.1168	0.3766
	C7	*Y* = − 0.01101*X + 8.245	0.0972	0.01106	0.1277	0.3217
X = Cobb Y = D2	C3	*Y* = 0.02339*X + 9.667	0.1213	0.01877	0.2167	0.2156
	C4	*Y* = − 0.01651*X + 9.601	0.0837	0.01927	0.2225	0.3937
	C5	*Y* = 0.01121*X + 9.967	0.0580	0.01892	0.2184	0.5548
	C6	*Y* = 0.02126*X + 10.07	0.1027	0.02019	0.2331	0.2948
	C7	*Y* = − 0.01897*X + 10.32	0.0867	0.02137	0.2467	0.3767
X = Cobb Y = SSA	C3	*Y* = 0.1167*X + 101.6	0.1350	0.08398	0.9695	0.1676
	C4	*Y* = 0.03185*X + 100.2	0.0326	0.09564	1.104	0.7398
	C5	*Y* = − 0.07320*X + 91.17	0.0953	0.07501	0.8659	0.3313
	C6	*Y* = 0.03906*X + 88.02	0.0467	0.08191	0.9456	0.6345
	C7	*Y* = 0.1238*X + 95.06	0.1376	0.08738	1.009	0.1594

* means multiply by

PH = pedicle height; D2 = the distance from the starting point to the lowest point; SSA = sagittal section angle

## Discussion

The current study focused on the shape of the pedicle and used six parameters based on CT scanning to establish a coordinate system to guide cervical spine pedicle screw placement. First D1 and D2 were used to coordinate positioning in this system, to replace the inaccurate but traditional method which relies on landmarks (articular mass, inferior articular process of the cephalad vertebra, and lateral vertebral notch) for the SP for placement of the pedicle [1, 19–22]. Unlike D1, which has gained much attention and has been the subject of extensive study, D2 was considered first in this study and deemed worthy of more attention because its value is relatively fixed in all segments. No previously published study has combined these two parameters to determine the insertion point of the pedicle. We have previously used D2 in clinical practice and found that it can be easily measured via CT reconstruction preoperatively, and during pedicle screw placement from C3 to C7.

Given that most preoperative preparations for pedicle screw placement only focus on the narrowest PW to choose the appropriate width of the pedicle screw, PW is often measured clinically. In the current study there was a linear correlation between PW and the pedicle’s narrowest PH, which can be depicted by an equation. We can incorporate PW—the most common clinical pedicle data—into the equation, obtain PH, and further guide the choice of pedicle screw size. At the same time the sagittal plane of the pedicle has a larger fault tolerance space, which means that the offset of the pedicle screw in the sagittal plane is comparatively safer than that in the transverse plane.

After confirming the SP of the pedicle screw by D1 and D2, and choosing the appropriate pedicle screw based on PW and PH, the angle of pedicle screw placement can be guided by TSA and SSA. TSA can be accurately measured via CT scanning, which is also a common and necessary parameter in surgical planning [20, 23]. Moreover, TSA was related to the sequence of the vertebra. The lower the vertebral body the larger the TSA, and D1 will also be larger. There was a positive correlation between D1 and TSA, which may be explained as a right triangle effect. D1 can be considered the right-angle edge, and TSA the opposite angle. The larger TSA is, the longer D1 is. For this reason TSA and D1 were the parameters used to confirm the transverse orientation of the pedicle screw.

The correlations between PW and PH, and D1 and SSA can be explained by examining the development of the pedicle. As the spine gradually ossifies after chondrification at the 6th week of embryonic development, three main ossification centers play an important role; one in the centrum, and one each on either side of the vertebral arch. Longitudinal and latitudinal growth of the vertebral body accompany the growth, development, and movement of the whole body. At the same time, the second primary ossification center and the mechanical load should also be considered [24–26]. However, these were not the focus of the current study.

The aim of the present study was to facilitate more individualized and accurate pedicle screw placement. With respect to individualization, the first considerations in adults are differences in gender, height, and weight. Pedicles in males and individuals of greater heights and weights were larger than those of females and individuals of smaller heights and weights. This is due to the innate proportional development of individuals [3, 27, 28].

We considered SSA to be another essential parameter that is often overlooked relative to TSA. Routine CT examination is in the supine position, and a change to the prone position during the operation would inevitably lead to data changes with respect to the sagittal alignment of the cervical spine. Therefore, if the sagittal angulation is positioned in the horizontal plane accuracy will be affected [3, 29], and if the lower edge of the vertebral body is used for positioning it is difficult to grasp during the operation [30, 31]. In the current study SSA was associated with each vertebral body’s D2 parameter, which can be understood as the lamina line. It is not associated with the sequence of vertebrae, rather it can be more accurately determined via direct visualization during the operation. The above-described six parameters obtained via CT scanning in the supine position to guide screw placement are accurate, and will not change regardless of how the position changes. More importantly, they are not affected by lordosis or kyphosis, degeneration and hyperplasia, imbalance or deformity of the cervical spine.

Notably we do not advocate memorizing the dates of the pedicle’s size and angle. Instead, each pedicle of each patient must be carefully measured before surgery so as to obtain 6 parameters that can be individually and accurately applied to surgery.

The current study had some limitations. The size of the sample was relatively small, and it was restricted to Chinese adults. Data measurements are likely to differ in other races and in children, but notably the same measurement methodology could be used. Secondly, the primary aim of the study was to provide an effective aid to assist freehand pedicle screw placement, and it is not appropriate to rely solely on the measurement data to place screws mechanically. The experience of the surgeon and intraoperative feel are still very important. Lastly, although we could precisely fix the SP position and the entrance angle, the system needs convenient devices with which to apply the parameters during surgery. Therefore, we are currently conducting further studies aimed at developing a new locating device based on the system, and broader clinical application of the system.

## Conclusion

Our localization system based on six parameters derived from CT reconstruction, namely D1, D2, TSA, SSA, PW, and PH, contributes to improved understanding of pedicle anatomy and helps improve the accuracy of cervical spine pedicle screw placement regardless of cervical sagittal alignment. Considering the accuracy, ease of use, and low cost of the system, it is expected to be widely used in clinical practice.

## Data Availability

Yes.
